# Therapeutic effects of sleeve gastrectomy for non-alcoholic steatohepatitis estimated by paired liver biopsy in morbidly obese Japanese patients

**DOI:** 10.1097/MD.0000000000026436

**Published:** 2021-07-02

**Authors:** Eisuke Murakami, Takashi Nakahara, Akira Hiramatsu, Kei Morio, Hatsue Fujino, Masami Yamauchi, Tomokazu Kawaoka, Masataka Tsuge, Michio Imamura, Hiroshi Aikata, Kenichi Fudeyasu, Yuki Nakashima, Daisuke Iwaki, Daichi Jodai, Toshikazu Ohigashi, Yui Nishimura, Yusuke Minamoto, Akiko Nagao, Masayasu Yoneda, Yoshihiro Saeki, Kazuaki Tanabe, Hideki Ohdan, Kazuaki Chayama

**Affiliations:** a Department of Gastroenterology and Metabolism; b Liver Research Project Center; c Natural Science Center for Basic Research and Development, Hiroshima University, Higashi Hiroshima; d Division of Rehabilitation, Department of Clinical Practice and Support; e Department of Pharmaceutical Services; f Division of Nutrition Management; g Department of Endocrinology and Diabetic Medicine, Hiroshima University Hospital; h Department of Gastroenterological and Transplant Surgery, Graduate School of Biomedical & Health Science; i Collaborative Research Laboratory of Medical Innovation, Hiroshima University, Hiroshima; j Institute of Physical and Chemical Research (RIKEN) Center for Integrative Medical Sciences, Yokohama, Japan.

**Keywords:** liver biopsy, morbid obesity, non-alcoholic steatohepatitis, sleeve gastrectomy

## Abstract

Bariatric surgery has been reported to improve non-alcoholic steatohepatitis (NASH), which is a frequent comorbidity in morbidly obese patients. We performed a retrospective cohort study to estimate the therapeutic effect of sleeve gastrectomy (SG), the most common bariatric surgery in Japan, on obese patients with NASH by comparing the findings of paired liver biopsies.

Eleven patients who underwent laparoscopic SG for the treatment of morbid obesity, defined as body mass index (BMI) > 35 kg/m^2^, from March 2015 to June 2019 at Hiroshima University Hospital, Japan, were enrolled. All patients were diagnosed with NASH by liver biopsy before or during SG and were re-examined with a second liver biopsy 1 year after SG. The clinical and histological characteristics were retrospectively analyzed.

One year after SG, body weight and BMI were significantly reduced, with median reductions in body weight and BMI of—22 kg and –7.9 kg/m^2^, respectively. Body fat was also significantly reduced at a median of 13.7%. Liver-related enzymes were also significantly improved. On re-examination by paired liver biopsy, liver steatosis improved in 9 of the 11 patients (81.8%), ruling out of the pathological diagnosis of NASH. However, fibrosis stage did not significantly improve 1 year after SG. The non-alcoholic fatty liver disease activity score was significantly reduced in 10 of 11 patients (90.9%).

Pathological improvement or remission of NASH could be achieved in most morbidly obese Japanese patients 1 year after SG.

## 1 Introduction

Non-alcoholic fatty liver disease (NAFLD) is rapidly increasing in incidence and is one of the most prevalent chronic liver diseases in many modern countries today and in the future.^[1,2]^ NAFLD has also been reported to be associated with metabolic disfunction such as obesity, type 2 diabetes, and metabolic syndrome.^[3]^ Some patients with NAFLD who show hepatocyte ballooning and liver fibrosis are histologically diagnosed with non-alcoholic steatohepatitis (NASH), which is recognized to have potential to progress from NAFLD.^[4,5]^ NASH may lead to increased mortality or an increased incidence of advanced severe liver diseases, including hepatocellular carcinoma.^[6]^ Obesity has been reported to be one of the most prevalent global epidemics over the years and is closely related to both NAFLD and NASH.^[7–10]^ Obesity and increased body mass index (BMI) are associated with the risk of NAFLD development.^[11]^ In addition, morbid obesity, defined as a BMI >35 kg/m^2^, was reported to be associated with a high prevalence of pathological liver diseases, including NASH.^[12]^ Seki et al^[13]^ reported that 77.5% of obese Japanese patients requiring bariatric surgery were diagnosed with NASH by liver biopsy. Obese patients with NAFLD have been recommended to undergo lifestyle intervention treatment; however, although patients who succeeded in weight reduction showed favorable outcomes, the achievement rate was reported to be limited.^[4,14,15]^ Because no successful pharmacological treatment has been developed, there is a pivotal global need for the effective treatment of NAFLD comorbid with obesity.

Bariatric surgery is the most effective method for weight reduction in patients with severe obesity and is termed “metabolic surgery” because it improves obesity-related metabolic disorders.^[16–19]^ There have been many reports on the resolution of NAFLD after bariatric surgery.^[20,21]^ In several prospective studies, improvements in NAFLD or NASH were estimated by comparing the findings of intraoperative and follow-up liver biopsies after bariatric surgery.^[22–29]^ Among the surgical procedures of bariatric surgery, laparoscopic sleeve gastrectomy (SG) only started to be covered by insurance in 2014 in Japan, which is a simple procedure for the surgical resection of the fundus part of the stomach. SG has also been reported to be an effective treatment for NAFLD in obese Japanese patients.^[13,30,31]^ Nikai et al^[32]^ reported that most patients with NASH were improved by SG and no longer met the diagnostic criteria for NASH by a second liver biopsy. Although bariatric surgery undoubtedly has a curative potential for NASH in morbidly obese patients, current reliable reports may not sufficiently prove the effects on obese Japanese patients. The aim of this retrospective cohort study was to estimate the therapeutic effects of SG in patients with NASH by comparing histological and metabolic changes before and 1 year after SG based on paired liver biopsy.

## 2 Methods

### 2.1 Patients

**Figure 1:**
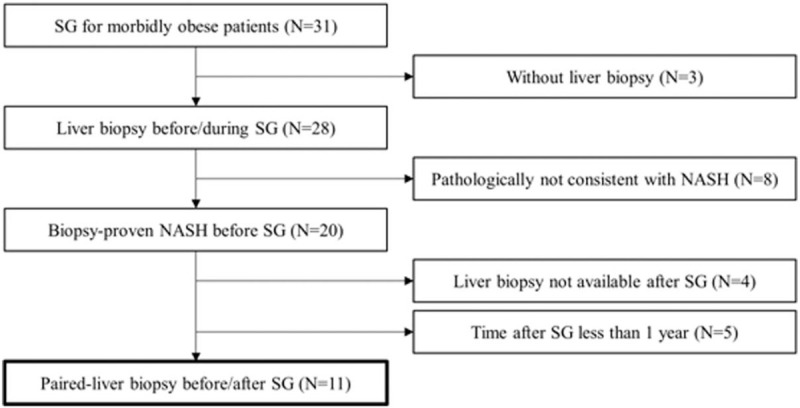
Flowchart of the enrollment of cases of paired- liver biopsy among morbidly obese patients treated with sleeve gastrectomy.

In Hiroshima University Hospital, laparoscopic SG was performed in 31 patients with morbid obesity between March 2015 and June 2020. Of these, 28 patients underwent liver biopsy before or during SG, and 20 patients were histopathologically diagnosed with NASH. Paired-liver biopsy (before and 1 year after SG) was performed in 11 patients. These 11 patients with biopsy-proven NASH were enrolled in this study (Fig. 1). Baseline clinical characteristics of the patients are summarized in Table 1. All patients received medication against at least one comorbid disease with a high percentage for each disease: 8 patients received medications for hypertension (72.7%), 7 for type 2 diabetes mellitus (63.6%), and 6 for dyslipidemia (54.5%). Concerning to the medication, 6 patients received angiotensin II receptor blocker (54.5%), 5 received metformin (45.5%), and 3 received thiazolidine (27.3%).

**Table 1:** Clinical characteristics of patients with NASH who underwent paired liver biopsy.

Factors	Values
Age, y	49 (32–57)^∗^
Men/Women	6 / 5
Observation period, mo	38.8 (15.6–53.0)^∗^
BMI at initial visit, kg/m^2^	46.2 (36.0–51.3)^∗^
Body weight at initial visit, kg	119.8 (81.5–139.7)^∗^
Comorbid diseases (with overlapping)	
Hypertension	8 [72.7%]
Type 2 diabetes mellitus	7 [63.6%]
Dyslipidemia	6 [54.5%]
Obstructive sleep apnea syndrome	4 [36.4%]

BMI = body mass index, NASH = non-alcoholic steatohepatitis.

∗ Values are shown as median (minimum–maximum) and number [percentage].

All patients satisfied the following criteria for SG established by Japanese insurance practice: morbid obesity (BMI > 35 kg/m^2^), age between 18 and 65 years, and insufficient control by medical treatment for >6 months of ≥1 comorbidities (type 2 diabetes, hypertension, dyslipidemia, or obstructive sleep apnea syndrome). Our exclusion criteria were as follows: secondary obesity due to pituitary or endocrine disorder, poorly controlled mental disorder or alcoholism, serologically positive of chronic viral hepatitis B or hepatitis C, clinically diagnosed by autoantibody positive autoimmune hepatitis or primary sclerosing cholangitis, and failure to lose 5% of the preoperative body weight after 6 months of nutritional intervention with a formula diet (Mirodiet, Sunny Health Co. Ltd., Japan) taken at least once a day. The preoperative and postoperative treatment plans or therapeutic indications for every patient were regularly discussed by a professional team named the Metabolic Care Unit, which consisted of several doctors, pharmacists, physical therapists, and dietitians. This study was conducted in accordance with the ethical principles of the Declaration of Helsinki and was approved by the Institutional Review Board of Hiroshima University (Ethical Committee for Epidemiology of Hiroshima University). Written informed consent was obtained from each patient after providing a detailed explanation of the study.

### 2.2 Liver histology

All enrolled patients underwent percutaneous liver biopsy under ultrasonic guidance or intraoperatively under laparoscopic control before or during SG. Formalin-fixed, paraffin-embedded liver sections were routinely stained with hematoxylin–eosin, silver reticulin, and Masson trichrome. All specimens were examined by an experienced pathologist blinded to the clinical data of the patients. The histological diagnosis of NASH was made according to the Matteoni classification. Grading and staging were performed according to Brunt classification, as previously reported.^[5,33]^ Briefly, steatosis was graded as follows: grade 1 (5%–33% of hepatocytes affected), grade 2 (34%–66% of hepatocytes affected), and grade 3 (>66% of hepatocytes affected). Necroinflammation was graded from 0 to 3 as follows: grade 0, absent; grade 1, octagonal ballooned hepatocytes with no or very mild inflammation; grade 2, ballooning of hepatocytes and mild-to-moderate portal inflammation, and grade 3 (intra-acinar inflammation and portal inflammation). Fibrosis was staged from 0 to 4 as follows: grade 0, absent; grade 1, perisinusoidal/pericellular fibrosis; stage 2, periportal fibrosis; stage 3, bridging fibrosis; and stage 4, cirrhosis. Histological findings were defined as NAFLD activity scores. The diagnostic procedure was the same as that described in our previous report.^[34]^

### 2.3 Clinical data and statistical analysis

Laboratory or physical data were collected at the initial visit, on the day of liver biopsy, before SG, every 3 months after SG, and 1 year after SG. The following data were collected: body weight, BMI, blood pressure, liver function test results including aspartate aminotransferase (AST), alanine aminotransferase (ALT), and gamma glutamyl transpeptidase (γ-GTP) levels; glucose tolerance estimated using the fasting blood glucose (FBG) and glycosylated hemoglobin A1c (HbA1c) levels; serum lipids, including cholesterol and triglyceride levels; and nutritional parameters such as albumin, hemoglobin, and body composition. Body composition was tested using bioelectrical impedance analysis (InBody 720; InBody Japan Inc., Tokyo, Japan). The clinical data and baseline laboratory data before SG and 1 year after SG are shown in Tables 1 and 2. Data were statistically analyzed on July 1, 2020. The differences in each factor before and after SG were examined for statistical significance using the Mann–Whitney *U* test. Statistical significance was set at *P* < .05. Statistical analysis was performed using IBM SPSS Statistics for Windows (version 22.0; IBM Corp., Armonk, NY).

**Table 2:** Comparisons of clinical parameters between before and 1 year after sleeve gastrectomy (SG).

Factors	Before SG^∗^	1 year after SG^∗^	*P* value
Albumin, mg/dL	4.1 (3.6–4.5)	4.2 (3.2–4.5)	N.S.
Hemoglobin, g/dL	14.1 (11.5–16.9)	13.3 (8.8–15.7)	N.S.
Platelet (×10^3^/mm^3^)	236 (123–369)	223 (117–408)	N.S.
AST, IU/L	30 (16–46)	18 (12–26)	<.05
ALT, IU/L	45 (22–66)	16 (7–44)	<.05
γ-GTP, IU/L	42 (11–129)	17 (9–41)	<.05
Systolic BP, mm Hg	136 (108–150)	128 (104–138)	N.S.
Diastolic BP, mm Hg	82 (68–98)	76 (68–88)	N.S.
FBG, mg/dL	120 (96–210)	98 (80–124)	<.05
HbA1c (%)	7.1 (5.6–10.0)	5.5 (4.5–7.3)	<.05
Cholesterol, mg/dL	187 (102–231)	172 (149–230)	N.S.
Triglyceride, mg/dL	116 (78–298)	75 (42–169)	N.S.
Fib-4 index	0.911 (0.450–2.325)	1.033 (0.559–2.220)	N.S.

γ-GTP = gamma glutamyl transpeptidase, AST = aspartate aminotransferase, ALT = alanine aminotransferase, BP = blood pressure, FBG = fasting blood glucose, Fib-4 = fibrosis-4, HbA1c = glycohemoglobin A1c, N.S. = not significant.

∗ Values are shown as median (minimum–maximum).

## 3 Results

### 3.1 Change of physical measurements

**Figure 2:**
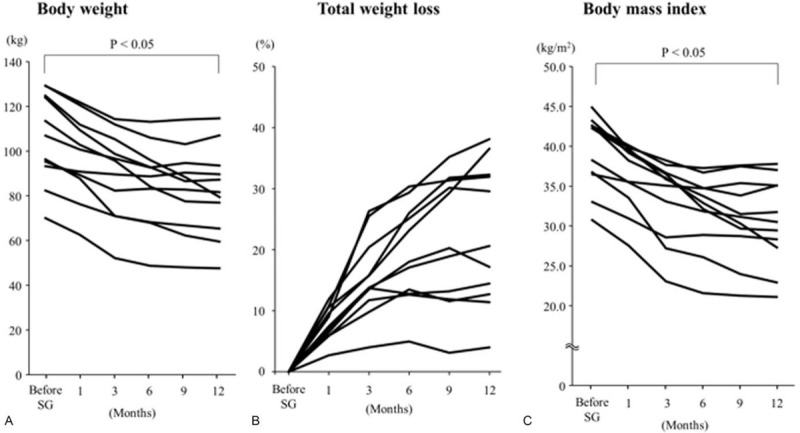
Change of physical parameters from baseline to 1 year after sleeve gastrectomy. (A) Body weight significantly decreased from baseline (*P* < .05). (B) Total body weight loss decreased from baseline with a median reduction of 80%. (C) Body mass index significantly decreased with a median reduction of –7.9 kg/m^2^ (*P* < .05).

**Figure 3:**
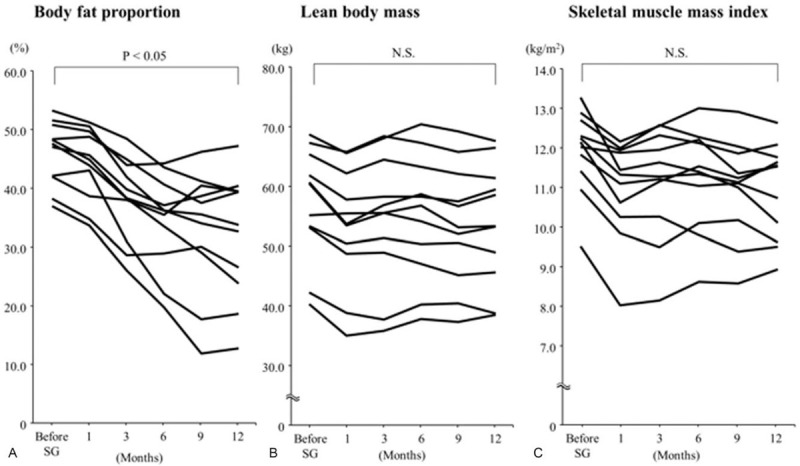
Change of body composition analysis parameters from baseline to 1 year after sleeve gastrectomy. (A) Body fat proportion significantly decreased from baseline (*P* < .05). (B) Lean body mass, calculated by body weight minus body fat weight, did not significantly change after sleeve gastrectomy. (C) Skeletal muscle mass index was not significantly change after sleeve gastrectomy. N.S. = not significant.

At 1 year after SG, all patients showed median reductions in body weight, ratio of total weight loss from baseline, and BMI of –22 kg, 80%, and –7.9 kg/m^2^, respectively. There was a significant difference in each parameter before and 1 year after SG (*P* < .05). Both body weight and BMI tended to decrease in the initial 6 months more rapidly than in the latter 6 months (Fig. 2A–C). The body composition test showed a significant reduction in body fat percentage (median, 13.7%) 1 year after SG (*P* < .05), whereas lean body mass and skeletal muscle mass index were successfully maintained without significant differences (Fig. 3A–C). All medications for comorbid diseases such as type 2 diabetes could be discontinued immediately after SG.

### 3.2 Change of laboratory data

Laboratory examination showed that the liver-related enzymes AST, ALT, and γ-GTP were significantly improved from baseline at 1 year after SG (*P* < .05, Table 2). The median reduction values of AST, ALT, and γ-GTP were –15, –25, and –23 IU/L, respectively. These variables tended to decrease rapidly in the first 3 months after SG. With respect to glucose tolerance, the median FBG and HbA1c values also significantly decreased to within the normal range 1 year after SG. The nutritional parameters albumin and hemoglobin were successfully maintained without significant differences between before and 1 year after SG. Serum lipid cholesterol and triglyceride levels did not show significant changes before or 1 year after SG.

### 3.3 Change of liver histology

**Figure 4:**
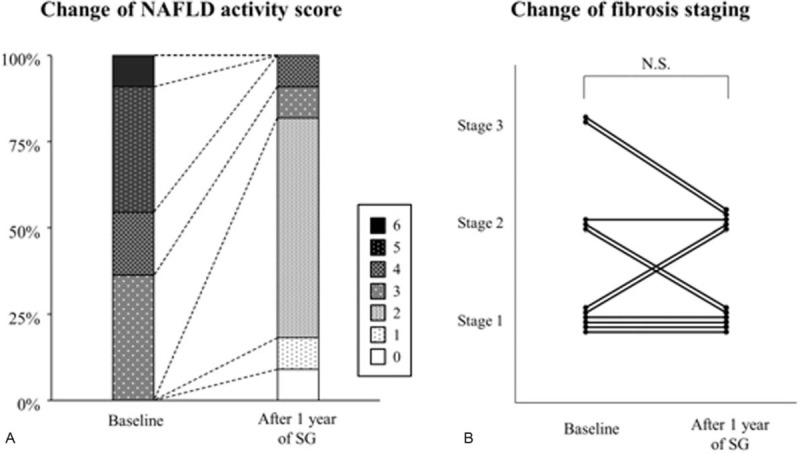
Change of liver histological findings of non-alcoholic steatohepatitis between before and 1 year after sleeve gastrectomy (SG). (A) The NAFLD activity score was significantly reduced in 10 of the 11 patients (90.9%). (B) Fibrosis stage was not significantly improved 1 year after SG: 4 patients improved by one stage, 5 had an unchanged stage, and 2 worsened for one stage. NAFLD = non-alcoholic fatty liver disease, N.S. = not significant.

**Figure 5:**
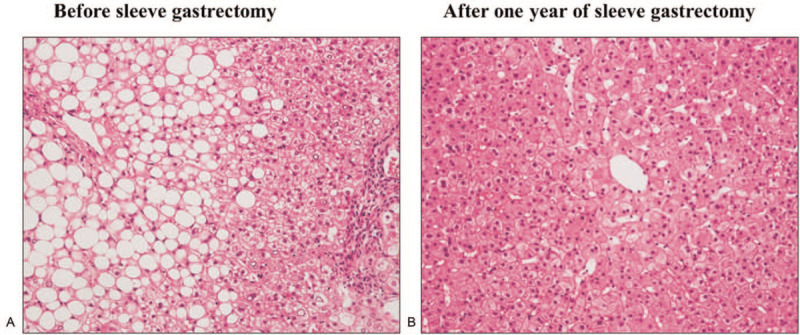
Representative histological findings in hematoxylin–eosin staining compared by paired liver biopsy between before and 1 year after sleeve gastrectomy (SG). (A) Before SG, liver specimen was diagnosed non-alcoholic steatohepatitis (NASH): Matteoni classification type 4, Brunt classification grade 3, stage 2, and non-alcoholic fatty liver disease activity score 6. (B) On re-examination after 1 year of SG, liver steatosis improved ruling out of the pathological diagnosis of NASH. The fibrosis stage improved stage 1. The non-alcoholic fatty liver disease activity score was reduced to 2.

All 11 patients underwent percutaneous liver biopsy to estimate histological improvement 1 year after SG. The pathological findings are summarized in Table 3. In 9 of the 11 patients (81.8%), NASH was no longer demonstrated because of improvements in liver steatosis 1 year after SG. The NAFLD activity score was reduced in 10 of the 11 patients (90.9%) and showed a significant difference between before and 1 year after SG (*P* < .05, Fig. 4A). However, the fibrosis stage did not significantly improve 1 year after SG: 4 patients improved by 1 stage, 5 had an unchanged stage, and 2 worsened by 1 stage (Fig. 4B). Representative pathological findings of liver biopsy before and 1 year after SG specimens stained with hematoxylin and eosin are shown in Fig. 5.

**Table 3:** Comparisons of liver histological findings of non-alcoholic steatohepatitis between before and 1 year after sleeve gastrectomy.

Case number	1	2	3	4	5	6	7	8	9	10	11
Matteoni classification	4/4	4/X	4/X	4/X	4/X	3/X	4/2	4/X	4/3	3/X	3/X
Brunt grading	2/2	2/X	2/X	1/X	2/X	3/X	1/X	2/X	3/1	1/X	2/X
Brunt staging	1/2	2/1	2/1	2/2	3/2	3/2	1/1	1/2	1/1	1/1	1/1
NAS	4/4	6/2	3/2	3/2	3/2	4/2	5/2	5/0	5/3	3/2	5/1
Steatosis	1/1	2/0	1/0	1/0	1/0	1/0	1/1	2/0	1/1	1/0	2/0
Lobular inflammation	2/2	2/1	1/1	1/1	1/1	2/1	2/1	1/0	2/1	1/1	1/1
Ballooning	1/1	2/1	1/1	1/1	1/1	1/1	2/0	2/0	2/1	1/1	2/0

Values were shown as before SG/1 year after SG. NAS = NAFLD activity score, X = not consistent with the criteria.

## 4 Discussion

Metabolic/bariatric surgery has been performed for morbid obesity worldwide owing to the rapid increase in the incidence of obesity in modern countries.^[17]^ The therapeutic effect of bariatric surgery is considered to involve not only weight loss but also improvement of prognosis in obesity-related diseases.^[16]^ NAFLD and obesity have been reported to be closely related and are emerging as public health problems in Asian countries, including Japan.^[7]^ The prevalence of NASH in Japanese patients with morbid obesity treated with bariatric surgery was higher than that reported in other countries.^[13,35]^ As recent modeling studies estimated a 49% increase in patients with NASH from 2016 to 2030 in Japan,^[2]^ an increase in the occurrence of NASH with morbid obesity is expected to be a Japanese medical issue that needs to be addressed. Our results may partially contribute to the establishment of a therapeutic option for treating NASH with morbid obesity in Japan in the future.

Since 2012, there have been several reports on the curative effects of bariatric surgery for NAFLD diagnosed by paired liver biopsy.^[22–27]^ These reports involved different numbers of patients, different observation times, and different surgical techniques for bariatric surgery. Among the surgical techniques, laparoscopic SG is one of the established methods of bariatric surgery, which is a simple procedure for surgical resection of the fundus part of the stomach. SG started to be covered by insurance in 2014 in Japan and is performed to treat morbidly obese patients in licensed institutions. The effectiveness of SG for Japanese patients with NASH has been reported by Nikai et al,^[32]^ who evaluated intraoperative liver biopsies and ultrasound-guided liver biopsies at 12 months after SG and observed improvement in 25 of the 28 patients. Our results seem to be comparable to this report in that 9 of the 11 patients (81.8%) achieved histological remission of NASH 1 year after SG in our study.

Liver fibrosis is known to be the most potent factor affecting mortality in patients with NAFLD.^[36]^ The effect of SG on decreasing liver fibrosis has been confirmed in several previous studies in morbidly obese patients with NAFLD.^[22,24,26,28,32]^ Contrary to these previous results, the fibrosis stage was not significantly reduced in patients with NASH 1 year after SG in our study (Figs. 4B and 5). In the same histological findings, neither perisinusoidal fibrosis nor periportal fibrosis also demonstrated significant reductions. The reasons for the discrepancy were considered to be related to the existence of 2 patients whose liver specimens demonstrated a one-stage worsening of fibrosis. These 2 patients experienced favorable body weight loss, but their comorbid diseases persisted for 1 year after SG: one had a late recurrence of type 2 diabetes and the other had mild hyperuricemia and hypertriglyceridemia. It cannot be ruled out that the persistence of comorbid diseases had some effects on the progression of fibrosis. Non-invasive liver fibrosis scores have been reported to be useful markers for NAFLD treated with bariatric surgery.^[20,37]^ In our study, no significant change in the fibrosis-4 index was calculated before and 1 year after SG, which was partially compatible with the histological findings (Tables 2 and 3). The other reasons might be related to the distribution of the enrolled patients in our study: relatively mild fibrosis (F0 or F1) was diagnosed in most of the patients before SG. Further accumulation of cases with moderate fibrosis is necessary to confirm the anti-fibrotic effect of SG observed in our study.

The present study had certain limitations, including the use of data generated from a single institution, small sample size, short observation periods to follow-up biopsy, and the possibility of sampling errors. Furthermore, there seemed to be some overestimation of the therapeutic effects based on our study protocol. However, our results may provide supportive data for future large-scale studies aimed at accurately identifying the effect of SG in morbidly obese Japanese patients with NASH as a comorbidity.

## 5 Conclusion

Eleven Japanese patients with morbid obesity comorbid with NASH and treated with SG were analyzed by paired liver biopsy performed before and after surgery. Pathological improvement or remission of NASH can be achieved in most patients 1 year after SG.

## Acknowledgments

The authors thank the hospital staff for their expert advice and assistance in the activity of the metabolic care unit.

## Author contributions

**Conceptualization:** Kazuaki Chayama.

**Data curation:** Eisuke Murakami, Takashi Nakahara, Kei Morio, Hatsue Fujino, Masami Yamauchi, Tomokazu Kawaoka, Masataka Tsuge, Kenichi Fudeyasu, Yuki Nakashima, Daisuke Iwaki, Daichi Jodai, Toshikazu Ohigashi, Yui Nishimura, Yusuke Minamoto, Akiko Nagao, Yoshihiro Saeki.

**Formal analysis:** Akiko Nagao.

**Funding acquisition:** Eisuke Murakami.

**Investigation:** Kenichi Fudeyasu, Yui Nishimura, Yusuke Minamoto, Yoshihiro Saeki, Kazuaki Tanabe.

**Project administration:** Akira Hiramatsu.

**Resources:** Yuki Nakashima, Daisuke Iwaki, Yui Nishimura, Yusuke Minamoto, Akiko Nagao.

**Supervision:** Akira Hiramatsu, Michio Imamura, Hiroshi Aikata, Masayasu Yoneda, Kazuaki Tanabe, Hideki Ohdan, Kazuaki Chayama.

**Writing – original draft:** Eisuke Murakami.

**Writing – review & editing:** Akira Hiramatsu.
